# Ultrasonographic characteristics of thyroid metastasis from clear cell renal cell carcinoma: A case report

**DOI:** 10.1097/MD.0000000000023070

**Published:** 2020-11-06

**Authors:** Peng Tian, Wenyan Du, Xiaoxi Liu, Wenzhe Xu, Xiaoyue Rong, Zekai Zhang, Yanzhen Wang

**Affiliations:** a Department of Ultrasonic; b Department of Science and Education, Zibo Central Hospital, Zibo, Shandong Province, China.

**Keywords:** clear cell renal cell carcinoma, thyroid metastasis, ultrasonography

## Abstract

**Introduction:**

Thyroid metastasis from clear cell renal cell carcinoma (ccRCC) is a very rare condition, and its ultrasonographic characteristics have not been summarized in the literature. We herein report a case of thyroid metastasis from ccRCC that occurred 11 years after the surgery and the ultrasonographic characteristics of it are described.

**Patient concerns:**

A 57-year-old male patient was admitted to our hospital in September 2018 due to discomfort in the neck. No other abnormalities were found during laboratory examination of thyroid function. The previous medical history of the patient included a right nephrectomy for the treatment of ccRCC in June 2007.

**Diagnosis:**

Ultrasound examinations revealed multiple thyroid nodules. After nephrectomy, there was no obvious abnormality in the right renal area. Computed tomography (CT) showed an oval lesion with slightly lower density in the right lobe of the thyroid, and the patient was initially diagnosed with nodular goiter.

**Interventions:**

Bilateral partial thyroidectomy under general anesthesia was conducted. Intraoperative frozen pathological examination showed clear cell carcinoma in the right lobe of the thyroid gland. Therefore, total thyroidectomy and lymph node dissection in the central neck area were performed.

**Outcomes:**

The patient underwent surgical treatment and recovered successfully. The patient was followed up for 2 years with no further metastasis.

**Conclusion:**

Ultrasound examination is a safe and convenient screening method. For patients with a renal malignant tumor, if the ultrasound image of thyroid nodule is found to have the characteristics of malignant tumors, the occurrence of metastasis of renal cancer to the thyroid should be highly suspected. Core needle biopsy (CNB) and histopathological diagnosis should be conducted subsequently for early diagnosis.

## 1 Introduction

The thyroid is the biggest endocrine gland in the human body, and the incidence of tumors associated with the thyroid gland is the highest among all the endocrine tumors. However, metastatic thyroid tumors accounts for about 1.4% to 3% of all thyroid malignancies. Besides, autopsy findings show that the incidence of thyroid tumors was as high as 1.9% to 24%.^[1]^ According to the previous studies, the 3 leading primary cancers included lung cancer (45.7%), esophagus cancer (25.7%), and breast cancer and renal cancer (5.7% for both cancers).^[2]^ To our knowledge, thyroid metastasis from ccRCC is uncommon, and only very few cases have been reported till date. Therefore, the ultrasonographic characteristics of thyroid metastasis from ccRCC are still lacking. We herein retrospectively analyzed the ultrasound imaging characteristics of thyroid nodules between 2012 and 2018 in a case with ccRCC who received surgical treatment. Also, the ultrasound imaging characteristics of thyroid metastasis from ccRCC from previous literature are discussed. These findings could provide insights on ultrasound diagnosis of thyroid metastasis from ccRCC.

## 2 Case report

**Figure 1:**
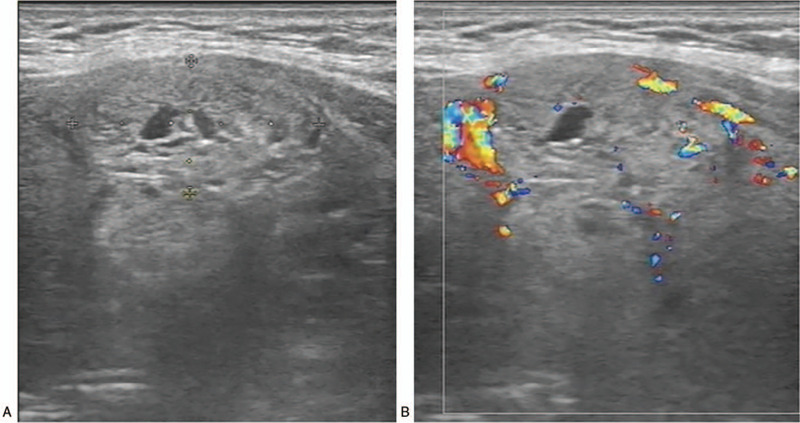
Ultrasound images of the patient in 2015. (A) A 2-dimensional image showing the size of the nodule of about 2.5 × 1.3 cm, with small patchy fluid sonolucent area was found in the lesion. (B) CDFI showed circular blood flow signals around the nodule and poor blood flow signals inside the nodule.

**Figure 2:**
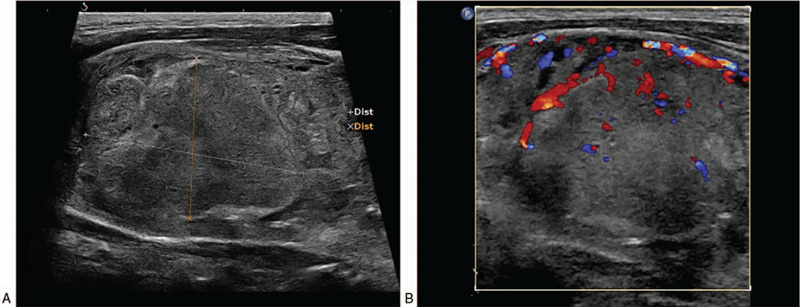
Ultrasound images of the patient in 2018. (A) A 2-dimensional image showing the size of the relatively big nodule of about 5.6 × 3.7 cm in the right lobe. (B)CDFI shows rich blood flow signals around and inside the nodule.

**Figure 3:**
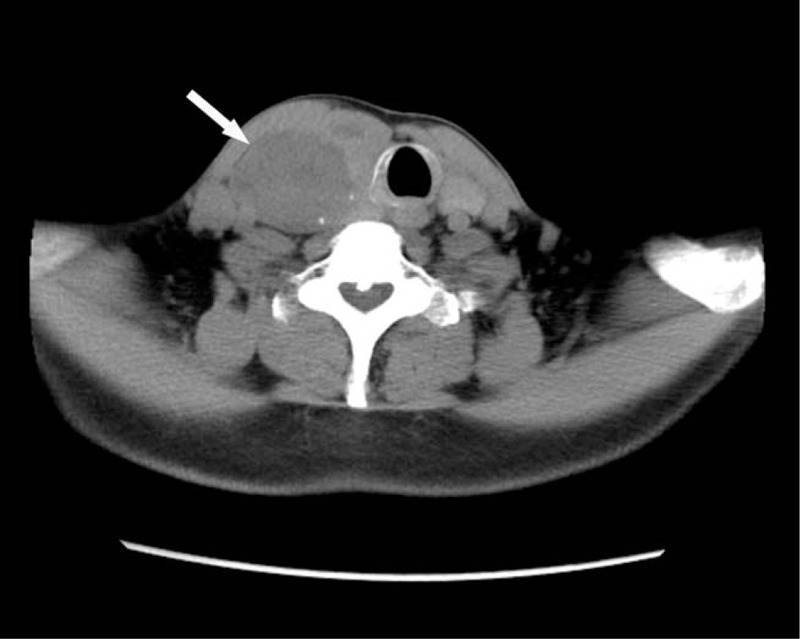
CT image in 2018. Arrow shows the lesion, with sand-like calcification around the lesion.

A 57-year-old male Han Chinese patient was admitted to our hospital in September 2018 due to neck discomfort. Physical examination on admission showed a local bulge on the right side of the neck, while the trachea was shifted to the left. Clinical examination revealed a large lump on the right lobe of the thyroid gland, which was approximately 5 × 4 cm in size. His previous medical history showed that he was admitted to the hospital in June 2007 due to painless hematuria and was diagnosed with right renal carcinoma. He then underwent radical resection of the right renal carcinoma. Postoperative pathological examination showed a moderately differentiated right ccRCC (stage III), and the patient did not receive further treatment after the operation. The re-examination of kidneys was done every year through ultrasound, which showed no abnormal echo in the right renal fossa. Ultrasound examination in July 2012 in our hospital showed the presence of multiple thyroid nodules, and the size of the largest nodule was about 1.2 × 0.6 cm, which was present in the right lobe with a clear boundary and homogenous echo. The patient refused the treatment because he was asymptomatic. Color ultrasound examination of thyroid in March 2015 revealed that the size of the largest nodule with clear boundary in the right lobe was about 2.5 × 1.3 cm, but the echo was heterogeneous, and small patchy fluid sonolucent area was found in the lesion. Color Doppler flow imaging (CDFI) showed circular blood flow signals around the nodule and poor blood flow signals inside the nodule (Fig. 1). So, re-examination by ultrasound was suggested due to his previous medical history. The results of the ultrasound examination conducted in September 2018 showed an evident increase in the volume of the right thyroid lobe, and the shape of the right lobe also remained abnormal. The thickness of the right lobe was 4.6 cm. Multiple hypoechoic nodules were detected in the thyroid gland, and the size of the largest nodule was about 5.6 × 3.7 cm (in the right lobe, and was merged with several nodules), in which the boundary was unclear, and the internal echo remained heterogeneous. In addition, dotty calcification and irregular fluid sonolucent area were also found. The results of CDFI showed relatively rich blood flow signals inside and around the nodule (Fig. 2). CT showed an oval lesion with slightly lower density in the right lobe of the thyroid, and the area of the maximum cross-section was about 5.1 cm × 4.3 cm. The density inside the lesion was slightly uneven, with a mean CT value of about 30 Hu, and multiple sand-like calcifications were found (Fig. 3). Laboratory examinations revealed serum free tri-iodothyronine (T3) of 5.45 pmol/l, serum-free tetraiodothyronine (T4) of 7.72 pmol/l, serum thyrotropin (TSH) of 1.01 uIU/ml, thyroglobulin antibody (TGA) of 0.00 IU/ml, thyroid peroxidase antibody (TPOA) of 0.70 IU/ml and parathyroid hormone (PTH) of 23.50 pg/ml.

**Figure 4:**
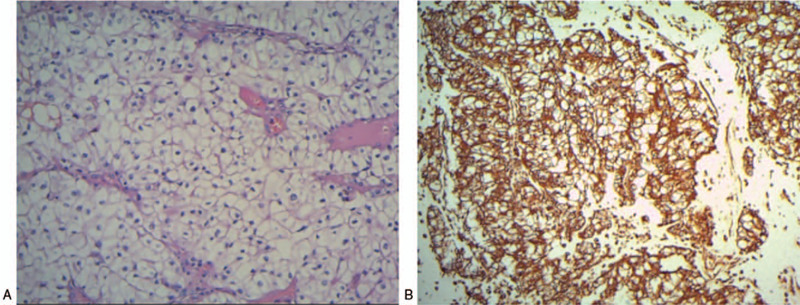
Pathological images of the thyroid tissues obtained during the surgery in 2018. (A) Hematoxylin-eosin (HE) staining (magnitude: 20 × 10). (B) Immunohistochemistry shows CD10 (+) (magnitude:10 × 10).

As thyroid metastasis from ccRCC is very rare, and the patient had a long history of a thyroid nodule, thyroid metastasis was not considered, and so the patient was initially diagnosed with nodular goiter. The patient chose surgical resection and refused a preoperative fine needle aspiration cytology (FNAC) examination to confirm the pathology. Bilateral partial thyroidectomy under general anesthesia was conducted. Intraoperative frozen pathological examination showed clear cell carcinoma in the right lobe of the thyroid gland, which was surrounded by benign thyroid tumors and accompanied by borderline tumors in the left lobe. Therefore, total thyroidectomy and lymph node dissection in the central neck area was conducted. The postoperative pathological examination showed clear cell carcinoma in the right thyroid tissue (tumor size: 4 × 3. 5 × 3.5 cm), and nodular goiter accompanied with non-typical follicular adenoma around the clear cell carcinoma and no metastasis in one of the lymph nodes in the central neck area of the left lobe. Immunohistochemistry revealed CKAE1/AE3 (+); Vimentin (+); CD10 (+); CK8/18 (partially +); CK7 (-); CK19 (-); Galectin-3 (-); CD117 (-); RCC (-); TG (-); CT (-); PTH (-); P53 (+), and S of 8%; and Ki-67 (+) and S of 15% (Fig. 4). According to immunohistochemistry findings and previous medical histories, the patient was confirmed with the diagnosis of thyroid metastasis from ccRCC. The patient recovered well from the operation and was advised to receive targeted therapy in the Oncology Department. However, the patient refused further treatment considering financial issues. The patient was followed up for 2 years and had not developed any additional metastasis. This case report was approved by the Ethics Committee of Zibo Central Hospital and the patients informed written consent.

## 3 Discussion

Renal cell carcinoma (RCC) is the most common renal malignancy, and ccRCC is the most common type, accounting for about 60% to 85% of all RCC. About 90% of all distant metastatic renal carcinomas are considered as metastases from ccRCC.^[3]^ However, thyroid metastasis from RCC is very rare in clinically. Lack of typical signs and symptoms usually would lead to a missed diagnosis of metastatic thyroid tumors. Most of the patients with metastatic thyroid tumors generally show no symptoms. Sometimes, patients might seek medical services because of dysphagia, wheezing, hoarseness, palpable lumps or enlarged lymph nodes in the neck. At present, ultrasound examination is regarded as the most common method for thyroid disorders,^[4]^ and has various advantages such as convenience, low cost, radiation-free, and high diagnostic accuracy. Thyroid nodules are discovered in some patients when undergoing medical examinations. Similarly, thyroid nodules, in this case, were reported during an ultrasound examination performed in July 2012. However, no further examination or treatment was conducted due to the lack of symptoms.

Thyroid imaging reporting and data system (TI-RADS) grading is recommended for ultrasound examination of thyroid nodules in several aspects, such as the boundary of thyroid nodules, internal echo, the existence of capsule, shape regularity, distribution of blood flow, aspect ratio, and microcalcifications, which is of definitive diagnostic value in distinguishing malignant thyroid tumors from benign ones.^[5]^ Most of the thyroid malignant tumors are primary tumors, in which papillary thyroid carcinoma is the most common type.^[6]^ Ultrasound images of papillary thyroid carcinoma mainly show single lesions with unclear boundaries, no capsule, irregular shape, the aspect ratio of larger than 1, hypoecho, and poor blood flow. Such single lesions can be accompanied by sand-like fine calcifications. Previous studies of ultrasound imaging characteristics of thyroid metastasis from ccRCC are summarized (Table 1).^[7–11]^ The results showed that the thyroid metastasis from ccRCC generally appeared as solid or solid-cystic nodules on ultrasound images, which could be single or multiple, mostly occurring in irregular shapes with heterogeneous internal echo, and were generally accompanied with calcifications or liquidation. Besides, CDFI generally showed rich blood flow signals and high-resistive Doppler waveforms. Based on these ultrasound imaging characteristics, it was speculated that these ultrasound images could be associated with biological features of ccRCC. For instance, ccRCCs are rich in capillaries and blood sinus, which are thus associated with a high risk of hemorrhage, necrosis, cystic degeneration, and calcification.^[12]^ This case showed an increase in the number of thyroid nodules during ultrasound examination in September 2018, in which some nodules were merged, while some nodules showed liquefied areas, dotty calcifications in addition to rich but tangled blood flow. These were consistent with the ultrasound imaging characteristics listed in Table 1. The ultrasound images of this case were similar to that of nodular goiter, and hence have a high risk for misdiagnosis. The findings of this case report were in agreement with the previous research reported by Krzysztof et al,^[13]^ which showed difficulty in distinguishing simple nodular goiter from the metastasis thyroid tumor of ccRCC by ultrasound imaging. The metastatic lesion is covered by nodular goiter, leading to difficulty in being discovered.

**Table 1:** Ultrasound images of thyroid metastasis from renal clear cell carcinoma reported in previous literatures.

Case number	First Authors (PubMed Unique Identifier, year)	Number of reported cases	Ultrasonographic features
1	Song OK et al^[7]^ (27956733,2017)	9	Single lesion (33.3%), multiple lesions (66.7%), solid echo pattern (66.7%), clear boundary (88.9%), regular shape (33.3%), with calcification (22.2%), and with rich blood flow (100%)
2	Cilengir AH et al^[8]^ (28096905,2016)	1	Single lesion, solid, hypoecho, with clear boundary, irregular shape, lobulated, with microcalcification, and with rich blood flow
3	Ramírez-Plaza CP et al^[9]^ (25827295,2015)	1	Multiple lesion, solid-cystic, unclear boundary, irregular shape, without calcification, and with rich blood flow in the solid region
4	Gheorghiu ML et al^[10]^ (31258806,2016)	1	Single lesion, solid-cystic, unclear boundary, regular shape, and with rich blood flow in the solid region
5	Wada N et al^[11]^ (15912297,2005)	1	Multiple lesions, solid, hypoecho, clear boundary, irregular shape, with calcified plaque, and with poor blood flow

Although the ultrasound images of thyroid metastasis from ccRCC show certain characteristics, it is still very difficult to distinguish primary thyroid carcinoma from metastatic tumors by ultrasound examinations. Ultrasound-guided FNAC of a thyroid nodule is a simple method with reasonable cost, which could achieve a qualitative diagnosis of most of the thyroid lumps. So FNAC is widely used as a screening tool for metastatic thyroid cancer.^[14]^ Although FNAC was highly accurate in the pathological diagnosis of metastatic thyroid cancer, Chung et al^[15]^ conducted a retrospective analysis of metastatic thyroid tumor and found that the false-negative rate of FNAC was 28.7%. Metastatic thyroid cancer is usually characterized by abundant vessels, which leads FNAC samples more likely to be contaminated with blood and makes the cytological diagnosis more difficult. However, enough tissue samples could be obtained for histopathological diagnosis by CNB, which makes CNB more sensitive and less false negative compared to FNAC. Immunohistochemical diagnosis is a valuable method for diagnosing thyroid metastasis from ccRCC.^[16]^ The metastatic tumor cells are negative for thyroglobulin, calcitonin, or TTF-1, but positive for CD10 and vimentin, which are in agreement with the postoperative immunohistochemistry report in our case. CT examination of thyroid nodules also has certain diagnostic value in differentiating benign lesions from malignant ones. However, CT images of metastatic thyroid carcinoma are similar to that of primary malignant thyroid tumors. So CT images are therefore non-specific.^[17]^ CT manifestations of malignant thyroid tumors vary, which include the single nodular type, the multiple nodular type, and the thick-walled cystic type. With CT examination, metastasis lesions may show the site of calcification. CT scanning in our case showed a single nodule accompanied by calcification, which was in agreement with the CT manifestations of malignant thyroid carcinoma. Previous studies^[18]^ have reported that metastatic thyroid tumors are not capable of iodine absorption, and thus ^131^I scanning of metastatic thyroid carcinoma shows cold nodules. Therefore, radionuclide scanning has showed certain diagnostic value in identifying metastatic thyroid tumors from primary thyroid carcinomas.

Most thyroid metastases of ccRCC are asymptomatic neck lumps, which usually appears 2∼7 years, or even 24 years after the operation.^[19]^ The case in this study was diagnosed with thyroid metastasis 11 years after the operation. Therefore, life-long follow-up is suggested for renal carcinoma patients who receive surgical treatment. Meanwhile, ultrasound examinations of the thyroid gland should be routinely conducted. In the cases with suspicious nodules, ultrasound-guided aspiration and biopsy are supposed to be conducted as early as possible to clarify the pathological type. Surgical resection should be conducted as early as possible for patients diagnosed with metastatic carcinomas.^[20]^

## 4 Conclusions

Ultrasound images of metastatic thyroid tumors arising from ccRCC present as solid or solid-cystic mass with clear boundaries, irregular morphology, calcification and plenty of blood flow signals. In the event that these typical characteristics are found in the thyroid ultrasound examination, combining with a history of RCC, thyroid metastasis of ccRCC should be highly suspected, which can be confirmed by CNB and histopathological diagnosis subsequently. Ultrasound is a safe and convenient initial examination, and the above-mentioned characteristic features on ultrasonography can be recognized as the clues to a correct diagnosis.

## Acknowledgments

The authors have no acknowledgments to disclose.

## Author contributions

**Data curation:** Wenyan Du, Xiaoxi Liu, Wenzhe Xu, Xiaoyue Rong, Yanzhen Wang.

**Formal analysis:** Peng Tian, Xiaoxi Liu, Xiaoyue Rong, Yanzhen Wang.

**Investigation:** Wenyan Du, Wenzhe Xu, Xiaoyue Rong, Zekai Zhang.

**Methodology:** Wenyan Du, Zekai Zhang, Yanzhen Wang.

**Project administration:** Peng Tian, Yanzhen Wang.

**Resources:** Peng Tian, Wenyan Du, Xiaoxi Liu, Zekai Zhang, Yanzhen Wang.

**Supervision:** Xiaoxi Liu.

**Validation:** Yanzhen Wang.

**Visualization:** Yanzhen Wang.

**Writing – original draft:** Peng Tian.

**Writing – review & editing:** Yanzhen Wang.
